# Feasibility and impact of *Creciendo Sanos*, a clinic-based pilot intervention to prevent obesity among preschool children in Mexico City

**DOI:** 10.1186/1471-2431-14-77

**Published:** 2014-03-20

**Authors:** Gloria Oliva Martínez-Andrade, Elizabeth M Cespedes, Sheryl L Rifas-Shiman, Guillermina Romero-Quechol, Marco Aurelio González-Unzaga, María Amalia Benítez-Trejo, Samuel Flores-Huerta, Chrissy Horan, Jess Haines, Elsie M Taveras, Ricardo Pérez-Cuevas, Matthew W Gillman

**Affiliations:** 1 Unidad de Investigación Epidemiológica y en Servicios de Salud, Instituto Mexicano del Seguro Social, CMN Siglo XXI, Av. Cuauhtémoc 330, Colonia Doctores, Delegación Cuauhtémoc, México, D.F. 06720, México; 2 Obesity Prevention Program, Department of Population Medicine, Harvard Medical School/Harvard Pilgrim Health Care Institute, 133 Brookline Avenue, 3rd Floor, Boston, MA 02215, USA; 3 Department of Nutrition, Harvard School of Public Health, 677 Huntington Avenue, Boston, MA 02115, USA; 4 Community Health Department, Hospital Infantil de México Federico Gómez, Secretaria de Salud, Dr. Márquez No.162, Col. Doctores, Delegación: Cuauhtémoc, México D.F. 06720, México; 5 Department of Family Relations and Applied Nutrition, University of Guelph, 50 Stone Road East, Guelph, Ontario N1G 2W1, Canada; 6 Division of General Pediatrics, MassGeneral Hospital for Children, 55 Fruit Street, Boston, MA 02114, USA; 7 División de Protección Social y Salud, Banco Inter Americano de Desarrollo, Avenida Paseo de la Reforma Nº 222 Piso 11, Colonia Juárez, Delegación Cuauhtémoc, México D.F. 6600, México

**Keywords:** Obesity prevention, Intervention, Trial, Pediatrics, Primary care, Mexico, Preschool

## Abstract

**Background:**

Mexico has the highest adult overweight and obesity prevalence in the Americas; 23.8% of children <5 years old are at risk for overweight and 9.7% are already overweight or obese. Creciendo Sanos was a pilot intervention to prevent obesity among preschoolers in *Instituto Mexicano del Seguro Social* (IMSS) clinics.

**Methods:**

We randomized 4 IMSS primary care clinics to either 6 weekly educational sessions promoting healthful nutrition and physical activity or usual care. We recruited 306 parent-child pairs: 168 intervention, 138 usual care. Children were 2-5 years old with WHO body mass index (BMI) z-score 0-3. We measured children’s height and weight and parents reported children’s diet and physical activity at baseline and 3 and 6-month follow-up. We analyzed behavioral and BMI outcomes with generalized mixed models incorporating multiple imputation for missing values.

**Results:**

93 (55%) intervention and 96 (70%) usual care families completed 3 and 6-month follow-up. At 3 months, intervention v. usual care children increased vegetables by 6.3 servings/week (95% CI, 1.8, 10.8). In stratified analyses, intervention participants with high program adherence (5-6 sessions) decreased snacks and screen time and increased vegetables v. usual care. No further effects on behavioral outcomes or BMI were observed. Transportation time and expenses were barriers to adherence. 90% of parents who completed the post-intervention survey were satisfied with the program.

**Conclusions:**

Although satisfaction was high among participants, barriers to participation and retention included transportation cost and time. In intention to treat analyses, we found intervention effects on vegetable intake, but not other behaviors or BMI.

**Trial registration:**

ClinicalTrials.gov NCT01539070.

Comisión Nacional de Investigación Científica del IMSS: 2009-785-120.

## Background

At 71%, the combined prevalence of overweight and obesity among Mexican adults ≥ 20 years old is the highest in the Americas, surpassing the United States (69%) [1,2]. Though under-nutrition remains an important issue in Mexico among lower income groups, obesity affects all economic groups and is increasing at greater rates within low-income than high-income populations [3,4]. A 2013 systematic review of the prevalence of overweight and obesity among children in Latin America reported that prevalence in Mexico is among the highest; in 2012, 23.8% of children <5 years old were at risk for overweight and 9.7% already overweight or obese [2,5]. A focus on early prevention will be essential to address a public health challenge of this magnitude since much of overweight and obesity begins in early childhood and tracks into later childhood and adulthood [6]. Overweight and obesity have immediate and long-term health consequences for children. Obese preschool children can experience adverse psychological outcomes, and physical consequences include increased risk of chronic conditions (i.e., cardiovascular diseases and diabetes) [7].

### Study rationale

The increasing burden of obesity in Mexico and globally corresponds not only to changes in physical activity and diet (unhealthy, high-calorie foods are widely available and inexpensive), but may be in part due to the lack of efficacy and effectiveness of health system prevention programs, and, importantly, to the weakness of harmonization between industry interests and public health policies [8]. Research suggests that a low level of education and interest in health issues may limit compliance with recommendations provided through the health system [9-11]. Yet, primary healthcare is a potentially pivotal setting for prevention and management of childhood obesity in Mexico. In 2012, the Mexican Social Security Institute (IMSS, *Instituto Mexicano del Seguro Social*) had 57 million affiliates (insured and beneficiaries) [12]. Among children 0-9 years affiliated with IMSS, combined overweight and obesity prevalence was 35% in 2010 [13]. Most preventive care for young children is provided during well-child visits and immunizations, which offer opportunities for prevention, detection and referral for treatment of overweight and obesity [14]. Mexican clinical guidelines exist for the treatment and prevention of childhood obesity [15]; however, in the formative research for this pilot, we conducted interviews with 67 health personnel and 52 mothers of overweight and obese children and found no standardized approach to addressing childhood obesity or overweight detected at a medical appointment [16].

The *Creciendo Sanos* pilot is the first obesity-specific prevention intervention in IMSS clinics and the first to be formally studied through a randomized controlled trial. *Creciendo Sanos* developed and tested a new intervention based on motivational counseling to make changes in eating behaviors and physical activity. Few obesity prevention interventions in preschool children have been conducted in primary care settings [17-23], and the two published Mexican interventions that have addressed diet and physical activity in primary care settings were conducted among older, already obese youth [24,25]. The only program currently providing obesity prevention content for young children is ChiquitIMSS, a series of 5 interactive educational sessions offered to parents and children 3-6 years in IMSS clinics and child care centers. ChiquitIMSS covers 20 topics including vaccination, basic hygiene, accident prevention, addiction and domestic violence. Healthful nutrition and physical activity appear in only 1 of the 5 sessions, and the program’s obesity prevention impact has never been evaluated.

The objective of this study was to evaluate the feasibility and impact of a pilot intervention to prevent obesity in children 2 to <5 years old in Mexico City primary care clinics. The primary outcomes were parent report of child’s diet and physical activity at 3 months; secondary endpoints included diet and physical activity at 6 months and body mass index (BMI) at 3 and 6 months.

## Methods

### Design, setting and randomization

This pilot, cluster-randomized trial included 4 primary care clinics operated by IMSS. The project manager approached the directors of the 6 primary care clinics in Mexico City with the greatest proportion of preschoolers (approximately 5% children <5 years) to request their support for the project. Four clinics agreed to participate. Using a computer-generated randomization list designed by a US-based statistician with no connection to the intervention, we randomly assigned the 4 clinics 1:1 to either educational intervention sessions or usual care. Only after informed consent did participants learn of their treatment assignment.

Participants and study staff were blinded to intervention status at recruitment, screening and the baseline assessment. Study staff was not blinded to intervention status at the 3 and 6 month follow-up assessments.

### Inclusion and exclusion criteria

Participants comprised children aged 2 - <5 years of age whose BMI (calculated as weight in kilograms divided by height in meters squared) was above the median for age and sex (BMI z-score 0 - 3); who attended one of the participating IMSS clinics during the recruitment period for pediatric care, vaccination, or accompanying a family member; and whose parent or caregiver gave written consent to participate. Families were excluded if they planned to move residences or change primary care clinics during the study period; the child had motor limitations (e.g., physical disability or delay); or required a special diet by medical indication. Institutional Review Boards in the United States (Harvard Pilgrim Health Care Human Studies Committee) and Mexico (Comisión de Ética, Comisión Nacional de Investigación Científica, IMSS) approved the study.

### Protocol changes

In February, 2012 one clinic randomly assigned to the intervention arm declined to participate because there was insufficient space for study procedures. We substituted an additional clinic with a similar population before beginning recruitment but after the initial randomization. Recruitment began in intervention clinics in March, 2012. At that time children with BMI z-scores > 1.5 - 3 were eligible to participate. In August, 2012, when recruitment began in usual care clinics, owing to under-recruitment we expanded eligibility criteria to include children above the median BMI for age and sex (BMI z-scores 0-3). Recruitment continued in intervention and usual care clinics until October 2012. Despite the differing BMI criteria applied during the recruitment periods in intervention v. usual care clinics, BMI at baseline was similar between the two treatment groups, as described in the Results section.

### Screening and recruitment

**Figure 1:**
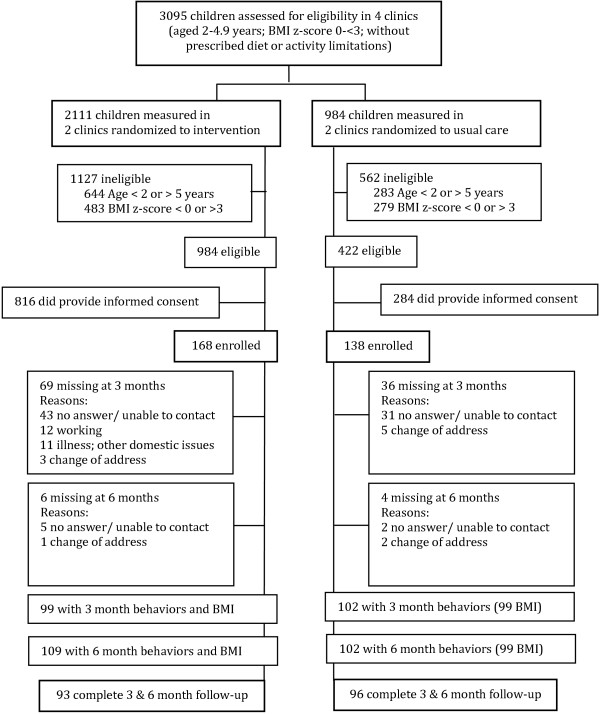
Recruitment and retention in the creciendo Sanos intervention.

From March through October, 2012, the research staff approached parents and caregivers of 3095 children in the waiting rooms of participating clinics (Intervention n = 2111; Usual care n = 984). Staff weighed and measured the children using a SECA 803 scale and SECA 213 mobile stadiometer and helped parents complete a baseline questionnaire to determine eligibility. If eligible, staff invited the family to participate and undergo informed consent. Of the children initially screened, 1406 (Intervention n = 984; Usual care n = 422) were eligible to participate and 306 (Intervention n = 168; Usual care n = 138) agreed to participate (Figure 1).

### Outcome measures

To assess children’s dietary intake at baseline and 3 and 6 month follow-up, staff assisted parents in completing a child Food Frequency Questionnaire (FFQ) adapted from the FFQ used to assess dietary intake among 1-4 year old children in the 2006 Mexican National Nutrition Survey [4]. We asked parents about the average number of days in the week or month the child consumed each food, the number of times daily the food was consumed on days when it was consumed, and the number of standard portions typically consumed each time the food was consumed. Responses for frequency of consumption were open-ended. From these responses we constructed grouped diet variables corresponding to food categories targeted in the educational intervention: sweet snacks (sugar-sweetened dairy, sugary cereal, cookies, sweet bread, cake, packaged pastries, caramel pops, candies and chocolates); fast food (hamburgers, pizza, hot dogs, quesadillas, fried tacos, French fries); savory snacks (packaged snack foods, corn or potato chips); fruit (orange, mango, papaya, watermelon, grapes, apple, banana); vegetables (chard, broccoli, jitomate [tomato], nopales [cactus], chayote [squash], spinach, lettuce, zucchini, carrot); sugar-sweetened beverages (soda, flavored milk, homemade [agua fresca] and packaged fruit drinks); and added sugar in beverages (teaspoons sugar or sweet flavoring added to milk, coffee, tea, or fruit juice).

To assess children’s physical activity at baseline and 3 and 6 month follow-up, we developed a physical activity and inactivity questionnaire. Staff assisted parents in reporting the average time the participating child spent in pre-specified active and sedentary activities during the week and on weekends. For each of the pre-specified activities parents reported time spent in open-ended response format. From these responses we derived total hours/week of physical activity composed of active play (e.g. running, jumping, walking, playing ball, playing in the park, biking, swimming, dancing), as well as total hours/week of screen time, composed of television, DVD/video, and video and computer games.

In order to calculate children’s BMI and age and sex specific BMI z-scores at baseline and 3 and 6 month follow-up, study staff assessed child’s height and weight using a SECA 803 scale and SECA 213 mobile stadiometer.

As part of the baseline interview, parents also reported their age, educational attainment and employment, marital status, family structure, and commute time and transportation costs to the clinic.

We assessed the feasibility of the intervention in the following ways: (i) scope, i.e., recruitment success, percentage of eligible families that accepted participation, and retention strategies; (ii) compliance, i.e. adherence in the intervention group measured through attendance at educational sessions, change in attendance following changes in the strategies of intervention delivery in response to low participation rates and participation in follow-up visits in both intervention and usual care; and (iii) acceptability, i.e., participant satisfaction with the intervention and cost and time involved in attending intervention sessions.

To assess scope and adherence, study staff kept detailed contact logs and field notes from recruitment and retention efforts (described below) for the intervention and usual care groups. From these, we calculated the total number of phone calls and drop-in home visits participants received to encourage attendance at study visits, as well as the number of educational sessions attended in the intervention group and whether sessions were individual or in group format. To assess acceptability, intervention parents attending ≥ 1 educational session completed a satisfaction survey at the 6 month follow-up visit. They rated their overall satisfaction with the intervention, how it had changed their attitudes towards their primary care clinic, how helpful each of the components had been for achieving behavioral changes, and time and cost involved in attending educational sessions at the clinic.

## Retention efforts

### Phone calls and home visits

Initially, we used phone calls to encourage attendance at the 3 and 6 month follow-up visits. When it became clear that retention was a challenge, we responded by adding home drop-in visits for participants who we could not contact by phone (94 participants at 3 months and 57 at 6 months, Additional file 1: Table S2). In cases where transportation, work schedules and domestic responsibilities prohibited the participant from coming in-person to the clinic we offered to complete the evaluations in the participant’s home (43 completed in-home at 3 months and 35 at 6 months, Additional file 1: Table S2).

### Incentives

As an additional retention strategy, we reimbursed participants for transportation costs to and from the clinic for follow-up evaluations at 3 and 6 months in the amount of 50 pesos (approximately $4). After completion of the 3 month visit both intervention and usual care participants received the children’s card game “memory” with pictures of foods. After completion of the 6 month visit both intervention and usual care families received a bound recipe book incorporating recipes intervention parents had written in the educational sessions.

## Intervention arms

### Usual care

Participants in clinics randomized to control received the usual standard of care. According to the existing clinical practice guide within IMSS, obese children may be referred to a nutritionist if the physician considers it appropriate or given general dietary advice by the attending physician. However, there are no standardized intervention programs specific to providing treatment to overweight or obese children at IMSS health care system. In the usual care clinics, we gave informed parents of their child’s height and weight and recommended they share the results with their physician in an upcoming medical consultation.

### Intervention

Creciendo Sanos was based on the High Five for Kids intervention [17], whose overarching conceptual model was the Chronic Care Model, which posits that changes in primary care to produce functional patient outcomes require changes for all members of the practice team as well as an informed and activated patient and family [26]. While High Five trained members of the existing clinical practice teams and enhanced electronic medical records, reorganizing the delivery of primary care was not feasible in this Mexican pilot. Thus, Creciendo Sanos focused on the part of the Chronic Care Model that informs and activates families. This approach innovates on a traditional health education approach. First, the intervention took place in clinic facilities to facilitate future integration with routine clinical practice and impress on participants the essential role of the nutrition and physical activity behaviors taught in maintaining children’s health. Second, as in High Five, educational sessions employed motivational interviewing and reflexive listening techniques to build rapport with participants and enhance self-efficacy, help parents recognize inconsistencies between actual and desired behaviors, and learn skills to reduce this dissonance, thus enhancing motivation for change. Components included de-emphasizing negative labeling, giving the parent responsibility for identifying which behaviors are problematic and modifiable, encouraging the parent to clarify and resolve ambivalence about behavior change, set concrete goals to initiate the change process and formally track progress towards goals [27-30]. Third, parent and child were actively engaged in practicing new knowledge during intervention sessions (e.g. playing active games, cooking healthy snacks, calculating the quantity of sugar and fat in processed foods from nutritional labels, and creating shopping lists). Finally, contextual barriers to change were addressed through a family-centered approach in which parents strategized collectively on how to engage all caregivers and family members in behavioral changes to achieve measurable progress. However, in Creciendo Sanos nurses and nutritionists were employed by the study rather than the clinic as in High Five, and participatory group workshops (rather than individual counseling sessions) were the primary means of delivering intervention content; individual make-up sessions were a secondary strategy.

### Educational workshops

The content of educational sessions drew from the High Five for Kids curriculum [17]. To adapt High Five to the needs of a Mexican pediatric population, we conducted semi-structured interviews with 67 health professionals and 52 mothers and caregivers of children with obesity or overweight. We identified key barriers to and facilitators of healthful nutrition (e.g., food purchase, preparation and serving of meals) and physical activity inside and outside the home (e.g., access to facilities and community safety). Qualitative analysis of the interviews with parents and caregivers is available elsewhere [16]. Adaptations for the Mexican context included group strategizing to help mothers get buy-in from other family members to implement changes in their homes and emphasizing foods and physical activities specific to Mexico City.

Before beginning the intervention, we tested the 6 session sequence with a group of 4 mothers who met the criteria for inclusion in the study but who were not participants in the final trial. Based on feedback during this initial testing, we replaced PowerPoint presentations with large canvas posters to better promote interaction and discussion among participants and included ample visual materials (e.g. food packaging) to cater to parents of all education levels. To ensure fidelity, a small group of study staff (nutritionist, nurse and health educator) administered all intervention sessions and completed all screening, baseline and follow-up assessments.

Participants randomized to intervention received a 6 week curriculum focused on obesity awareness and prevention. A trained nutritionist led diet, healthy growth and physical activity workshops, while a health educator led workshops on instilling healthy habits and routines in childhood. The nurse provided child care and developed relevant games and activities for children while parents attended the workshops.

**Figure 2:**
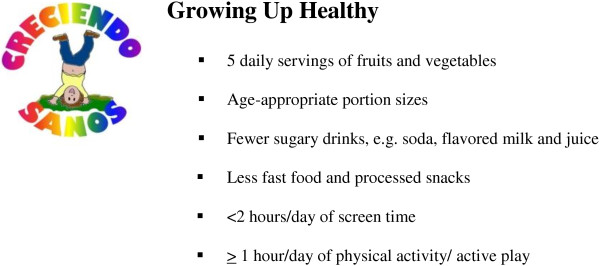
***Creciendo Sanos *** **Logo and Strategies.**

The weekly participatory workshops took place in a clinic classroom, auditorium or office. Each group was formed with approximately 10 parent-child pairs and completed as a cohort. We offered individual sessions in the clinic with the same content for participants who missed multiple group workshops or were unable to attend the weekly group workshops (19 of the 168 intervention parents received a total of 47 sessions delivered individually). At the start of the 6 week workshop we gave parents an illustrated manual with the main points of the curriculum, including the causes and consequences of childhood obesity, strategies for implementing behavioral changes and ideas for active play in the home, as well as educational content on each of the behavioral goals of the intervention, listed in Figure 2 and Additional file 1: Table S1. During the workshops, we provided childcare and active games for intervention children and their siblings.

The 6 educational sessions were 2 hours each: educational content on nutrition and physical activity (90 minutes) and socializing and building group rapport through activities like preparation and consumption of healthy foods on site (30 minutes). Sessions addressed appropriate portion sizes for children of different ages, the healthy eating plate, reading nutritional labels, planning healthy meals for the whole family, foods to increase and foods to limit, and strategies to increase active play and physical activity and reduce screen exposure. Each session had 4 components: i) Identification and analysis of obesity risk behaviors and proposals and family-specific strategies to change these behaviors; ii) Presentation and analysis of topics related to obesity and health consequences, e.g., monitoring child growth, healthful nutrition and/or physical activity habits, age-appropriate portion sizes, etc.; iii) Activities to support healthy nutrition and physical activity habits, e.g., preparing healthy snacks together or designing sample menus or healthy shopping lists; and iv) Suggestions for and practice of physical activities and active games for the home. The schedule for each session is in Additional file 1: Table S1.

### Statistical methods

To compare outcomes between intervention and usual care groups, we first examined baseline distributions of child and parent characteristics by intervention status. In intent-to-treat analyses, we used unadjusted and adjusted multivariate regression models to examine differences from baseline to 3 and to 6 months between the intervention and usual care groups. For continuous outcomes, we used linear regression models, and for dichotomous outcomes, we used logistic regression models. To account for clustering by practices, we performed generalized linear mixed models (PROC GLIMMIX in SAS version 9.3; SAS Institute Inc., Cary, North Carolina). All adjusted models included child age, change in age from baseline to 3 months, sex, BMI z-score, and total physical activity at baseline, and maternal education and occupation. We additionally adjusted for season at enrollment as a dichotomous variable because Intervention and Usual care recruitment were not completely congruent. We further adjusted each behavioral outcome model for the baseline value of the behavior of interest. We also conducted post-hoc stratified analyses to compare results by adherence defined by the number of educational workshops attended (0, 1-4, or 5-6).

To account for missing data, we performed multiple imputation for all 306 participants. All models are based on 306 participants. We used SAS imputations (Proc MI) to impute 50 values for each missing observation and combined multivariable modeling estimates using Proc MI ANALYZE in SAS version 9.3 (SAS Institute, Cary NC). An alternative approach, using only participants with all covariate and outcome data (complete case), yielded similar results.

To report on the feasibility of recruitment, retention and adherence, we calculated descriptive statistics from data in participant contact logs. For acceptability, we calculated descriptive statistics from data collected from the 6 month satisfaction survey completed by participants attending ≥ 1 educational session.

## Results

Figure 1 shows the participant flow for Creciendo Sanos: 65% of families completed 3 month follow-up (Intervention n = 99; Usual care n = 99) and 68% of families completed 6 month follow-up (Intervention n = 109; Usual care n = 99). Non-participation was greater in the intervention (75 (45%) of 168 participants) than in the usual care (42 (30%) of 138 participants) arm (Figure 1).

### Behavioral and BMI outcomes

At baseline, parents in intervention clinics were more likely to be homemakers (52% v. 46%) and less likely to have finished high school (50% v. 66%), Table 1. Children in intervention clinics were less physically active at baseline (24 v. 32 hours/week, Table 1). There were no group differences at baseline in other characteristics or health behaviors, including BMI: mean (SD) BMI was 17.3 (1.2) among intervention and 17.3 (1.1) among usual care children. WHO BMI z-scores were also similar among intervention (1.3 [0.8]) and usual care children (1.3 [0.7]), Table 1.

**Table 1:** **Baseline characteristics of participants in the *Creciendo Sanos *** **study overall and by intervention assignment**

	**Total**	**Intervention**	**Usual care**
**Characteristic**	**n = 306**	**n = 168**	**n = 138**
*Child characteristics*	**N (%)**		
Sex			
Male	161 (52.6)	87 (51.8)	74 (53.6)
Female	145 (47.4)	81 (48.2)	64 (46.4)
WHO BMI z-score category^1^			
Normal ≤1.0	135 (44.1)	79 (47.0)	56 (40.6)
Risk of overweight (>1.0- ≤2.0)	119 (38.9)	58 (34.5)	61 (44.2)
Overweight (>2.0)	52 (17.0)	31 (18.5)	21 (15.2)
	**Mean (SD)**		
Age, months	40.6 (10.0)	40.1 (10.1)	41.1 (9.9)
Height, meters	0.96 (0.07)	0.96 (0.07)	0.97 (0.07)
Weight, kg	16.22 (2.68)	16.07 (2.63)	16.40 (2.73)
BMI, kg/m^2^	17.3 (1.2)	17.3 (1.2)	17.3 (1.1)
WHO BMI z-score	1.27 (0.74)	1.28 (0.76)	1.26 (0.71)
*Parent characteristics*		**N (%)**	
Marital status			
Married	121 (39.5)	64 (38.0)	57 (41.3)
Domestic partnership	133 (43.5)	74 (44.0)	59 (42.8)
Divorced	5 (1.6)	2 (1.2)	3 (2.2)
Single	47 (15.4)	28 (16.8)	19 (13.8)
Employment			
Permanent	111 (36.3)	56 (33.4)	55 (39.9)
Contract	41 (13.5)	23 (13.9)	18 (13.0)
Unemployed	154 (50.2)	89 (52.7)	65 (47.1)
Occupation			
Homemaker	151 (49.2)	88 (52.1)	63 (45.7)
Professional	30 (9.9)	14 (8.5)	16 (11.6)
Office	15 (4.9)	7 (4.2)	8 (5.8)
Service	19 (6.2)	6 (3.6)	13 (9.4)
Factory	50 (16.5)	34 (20.5)	16 (11.6)
Retail	17 (5.6)	9 (5.4)	8 (5.8)
Other/Student	24 (7.8)	10 (5.8)	14 (10.1)
Educational attainment			
No schooling	1 (0.3)	0 (0.0)	1 (0.7)
Primary school	27 (8.9)	20 (12.0)	7 (5.1)
Junior high	103 (33.7)	64 (38.0)	39 (28.4)
High school	121 (39.3)	58 (34.4)	63 (45.3)
Professional school	38 (12.5)	20 (12.0)	18 (13.1)
Postgraduate	5 (1.7)	0 (0.0)	5 (3.6)
Other	11 (3.6)	6 (3.6)	5 (3.7)
	**Mean (SD)**		
Maternal age, years	29.4 (6.6)	29.3 (6.9)	29.5 (6.3)
Number of children in home	1.7 (0.8)	1.7 (0.8)	1.8 (0.8)
Transport cost to clinic, US $	1.27 (1.10)	1.27 (1.19)	1.27 (0.95)
Transport time to clinic, minutes	29.1 (17.9)	27.6 (16.0)	31.0 (19.8)
Number of educational sessions attended (0-6)		2.4 (2.6)	N/A
0 educational sessions attended		80 (47.6)	N/A
1-4 educational sessions attended		29 (17.3)	N/A
5-6 educational sessions attended		59 (35.1)	N/A
*Child behaviors*			
Sweet snacks, servings/week	21.0 (20.6)	20.7 (17.7)	21.3 (23.6)
Fast food, servings/week	1.9 (2.4)	2.0 (2.8)	1.7 (1.8)
Savory snacks, servings/week	1.3 (1.9)	1.2 (1.7)	1.5 (2.1)
Sugar-sweetened beverages, servings/week	15.5 (14.4)	15.6 (13.4)	15.3 (15.6)
Fruit, servings/week	24.1 (25.2)	21.3 (17.7)	27.5 (31.7)
Vegetables, servings/week	20.7 (17.5)	20.9 (15.0)	20.6 (20.1)
Added sugar in beverages, servings/week	11.0 (13.8)	11.1 (13.4)	10.8 (14.4)
Water, servings/week	22.0 (18.3)	20.8 (17.3)	23.5 (19.3)
Total physical activity, hours/week	27.4 (20.8)	24.0 (17.2)	31.6 (23.8)
Total sleep time, hours/day	11.0 (1.5)	11.3 (1.6)	10.7 (1.4)
Total screen time, hours/week	13.8 (11.1)	13.1 (11.2)	14.8 (10.9)

^1^WHO child growth standards: 2006.

Table 2 shows changes in our primary outcomes: diet and physical activity behaviors at 3 months. At 3 months, intervention v. usual care children increased vegetable consumption by 6.3 servings/week (95% CI, 1.8, 10.8). We did not detect changes in behavior at 6 months or BMI at either 3 or 6 months in intention to treat analyses. Some of the observed differences were fairly large, but given that this was a pilot intervention confidence intervals were wide and included null values. Examples included 3-month intervention v. usual care reductions in sweet snacks (-3.9 servings/week; 95% CI, -8.9, 1.1), sugar added to drinks (-2.2 servings/week; 95% CI, -8.4, 4.1) and total screen time (-1.6 hours/week; 95% CI, -4.4, 1.1). At 6 months these potential intervention effects were attenuated, Table 3.

**Table 2:** Change in BMI and behaviors from baseline to 3 months by intervention assignment

					**Intervention-control difference**	
		**Baseline**	**3 m**	**Change BL-3m**	**Unadjusted**	Adjusted*
**Outcome**		**Mean (SD)**	**Mean (SD)**	**Mean (SE)**	**Est (95% CI)**	**Est (95% CI)**
BMI, kg/m^2^	Intervention	17.3 (1.2)	17.1 (1.4)	-0.26 (0.01)	0.17 (-0.12, 0.46)	0.23 (-0.07, 0.54)
	Usual care	17.3 (1.1)	16.9 (1.2)	-0.44 (0.01)		
WHO BMI z-score	Intervention	1.28 (0.76)	1.12 (0.89)	-0.16 (0.01)	0.11 (-0.07, 0.30)	0.15 (-0.04, 0.35)
	Usual care	1.26 (0.71)	1.00 (0.78)	-0.27 (0.01)		
Sweet snacks, servings/week	Intervention	20.7 (17.7)	11.0 (11.4)	-9.7 (0.2)	-3.3 (-8.1, 1.4)	-3.9 (-8.9, 1.1)
	Usual care	21.3 (23.6)	14.9 (17.2)	-6.4 (0.3)		
Fast food, servings/week	Intervention	2.0 (2.8)	1.7 (1.6)	-0.4 (0.0)	-0.1 (-0.8, 0.5)	0.3 (-0.5, 1.1)
	Usual care	1.7 (1.8)	1.5 (1.8)	-0.2 (0.0)		
Savory snacks, servings/week	Intervention	1.2 (1.7)	0.6 (0.9)	-0.7 (0.0)	-0.1 (-0.6, 0.3)	-0.3 (-0.5, 0.0)
	Usual care	1.5 (2.1)	1.0 (1.2)	-0.5 (0.0)		
Sugar-sweetened beverages, servings/week	Intervention	15.6 (13.4)	9.6 (8.4)	-6.0 (0.2)	-2.8 (-6.5, 0.9)	-0.7 (-4.9, 3.4)
	Usual care	15.3 (15.6)	12.0 (11.8)	-3.2 (0.2)		
Fruit, servings/week	Intervention	21.3 (17.7)	21.4 (16.6)	0.1 (0.2)	2.8 (-17.2, 22.8)	-1.6 (-13.6, 10.3)
	Usual care	27.5 (31.7)	25.6 (24.8)	-1.9 (0.4)		
Vegetables, servings/week	Intervention	20.9 (15.0)	20.0 (17.1)	-0.8 (0.2)	4.5 (-1.0,10.1)	6.3 (1.8, 10.8)
	Usual care	20.6 (20.1)	15.1 (13.9)	-5.5 (0.2)		
Added sugar in beverages, servings/week	Intervention	11.1 (13.4)	7.1 (8.7)	-4.0 (0.2)	-2.3 (-6.3, 1.6)	-2.2 (-8.4, 4.1)
	Usual care	10.8 (14.4)	9.1 (12.0)	-1.7 (0.2)		
Water, servings/week	Intervention	20.8 (17.3)	20.2 (13.0)	-0.6 (0.2)	2.8 (-4.9, 10.4)	0.6 (-5.4, 6.5)
	Usual care	23.5 (19.3)	20.0 (15.9)	-3.5 (0.2)		
Total physical activity, hours/week	Intervention	24.0 (17.2)	26.9 (18.0)	2.9 (0.2)	-5.4 (-32.8, 22.0)	-11.8 (-29.1, 5.5)
	Usual care	31.6 (23.8)	41.1 (26.2)	9.5 (0.4)		
Total sleep time, hours/day	Intervention	11.3 (1.6)	11.0 (1.2)	-0.3 (0.0)	-0.2 (-0.7, 0.2)	0.2 (-0.2, 0.5)
	Usual care	10.7 (1.4)	10.7 (1.4)	-0.1 (0.0)		
Total screen time, hours/week	Intervention	13.1 (11.2)	10.3 (8.6)	-2.76 (0.1)	-0.8 (-3.8, 2.3)	-1.6 (-4.4, 1.1)
	Usual care	14.8 (10.9)	12.8 (10.7)	-1.97 (0.1)		

*Adjusted for child age, change in age from baseline to 3 months, sex, BMI z-score, and total physical activity at baseline; maternal education and occupation; and season of enrollment.

Behavioral models additionally adjust for baseline value of behavior of interest.

All models corrected for clustering by clinic.

**Table 3:** Change in BMI and behaviors from baseline to 6 months by intervention assignment

		**Change BL-6m**	**Unadjusted**	Adjusted*
**Outcome**		**Mean (SE)**	**Est (95% CI)**	**Est (95% CI)**
BMI, kg/m^2^	Intervention	-0.32 (0.01)	0.10 (-0.22, 0.42)	0.06 (-0.26, 0.37)
	Usual care	-0.43 (0.01)		
WHO BMI z-score	Intervention	-0.18 (0.01)	0.06 (-0.14, 0.27)	0.03 (-0.17, 0.23)
	Usual care	-0.25 (0.01)		
Sweet snacks, servings/week	Intervention	-10.2 (0.2)	-2.1 (-6.9, 2.6)	-2.3 (-5.8, 1.2)
	Usual care	-8.1 (0.3)		
Fast food, servings/week	Intervention	-0.6 (0.0)	-0.5 (-1.3, 0.4)	-0.3 (-1.3, 0.6)
	Usual care	-0.1 (0.0)		
Savory snacks, servings/week	Intervention	-0.8 (0.0)	-0.2 (-0.6, 0.3)	-0.4 (-0.7, -0.1)
	Usual care	-0.6 (0.0)		
Sugar-sweetened beverages, servings/week	Intervention	-4.0 (0.2)	-1.3 (-6.6, 4.1)	-1.2 (-4.8, 2.5)
	Usual care	-2.8 (0.2)		
Fruit, servings/week	Intervention	-3.0 (0.2)	5.6 (-17.2, 28.3)	0.2 (-11.7, 12.0)
	Usual care	-8.0 (0.4)		
Vegetables, servings/week	Intervention	-3.1 (0.2)	1.9 (-3.9, 7.6)	2.7 (-1.3, 6.7)
	Usual care	-5.0 (0.2)		
Added sugar in beverages, servings/week	Intervention	-1.7 (0.17)	-2.3 (-6.53, 1.9)	-1.4 (-6.9, 4.1)
	Usual care	0.6 (0.22)		
Water, servings/week	Intervention	-1.3 (0.21)	2.9 (-5.8, 11. 7)	1.5 (-3.3, 6.3)
	Usual care	-4.4 (0.2)		
Total physical activity, hours/week	Intervention	7.4 (0.3)	2.1 (-39.1, 43.3)	-4.3 (-32.9, 24.3)
	Usual care	6.7 (0.4)		
Total sleep time, hours/day	Intervention	-0.4 (0.0)	-0.2 (-0.6, 0.3)	0.1 (-0.2, 0.5)
	Usual care	-0.2 (0.0)		
Total screen time, hours/week	Intervention	-3.1 (0.1)	-1.2 (-4.9, 2.5)	-3.2 (-6.5, 0.2)
	Usual care	-1.9 (0.2)		

*Adjusted for child age, change in age from baseline to 6 months, sex, BMI z-score, and total physical activity at baseline; maternal education and occupation; and season of enrollment.

Behavioral models additionally adjust for baseline value of behavior of interest.

All models corrected for clustering by clinic.

In post hoc stratified analyses we did not observe intervention effects on behavioral endpoints or on BMI within subgroups defined by child age, sex, or BMI, or by maternal education or employment.

Some, but not all, intervention-control differences were of greater magnitude among intervention participants with higher levels of adherence, i.e., who more of the intended 6 educational workshops, Table 4. For sweet snacks, for example, the 80 intervention children who attended no workshops increased consumption by 0.9 servings/week (95% CI, -4.9, 6.7), whereas the 29 who attended 1-4 workshops decreased their intake by -5.5 servings/week (95% CI, -14.1, 3.0), and the 59 who attended 5-6 workshops decreased their intake by -9.1 servings/week (95% CI, -15.0, -3.2). We observed similar patterns for savory snacks and screen time among children of families who attended 5-6 of the 6 workshops, Table 4.

**Table 4:** **Intervention v. usual care 3-month behavioral outcomes in *Creciendo Sanos*, according to adherence to intervention**

	**Number of weekly educational sessions attended**		
**Outcome**	0 Adjusted* Est (95% CI)	1-4 Adjusted* Est (95% CI)	5-6 Adjusted* Est (95% CI)
BMI kg/m^2^	0.29 (-0.16, 0.73)	0.47 (-0.07, 1.01)	0.14 (-0.24, 0.52)
BMI z-score	0.19 (-0.10, 0.48)	0.29 (-0.06, 0.64)	0.10 (-0.15, 0.34)
Sweet snacks, servings/week	0.9 (-4.9, 6.7)	-5.5 (-14.1, 3.0)	-9.1 (-15.0, -3.2)
Fast food, servings/week	0.4 (-0.3, 1.2)	0.1 (-0.9, 1.0)	0.0 (-1.0, 1.0)
Savory snacks, servings/week	-0.1 (-0.5, 0.3)	-0.2 (-0.8, 0.4)	-0.4 (-0.8, -0.1)
Sugar-sweetened beverages, servings/week	0.5 (-4.2, 5.2)	-1.2 (-7.7, 5.4)	-1.6 (-6.0, 2.8)
Fruit, servings/week	-0.4 (-13.8, 12.9)	-2.0 (-17.7, 13.6)	-2.9 (-16.1, 10.4)
Vegetables, servings/week	7.2 (1.0, 13.3)	2.2 (-5.3, 9.7)	6.6 (1.7, 11.4)
Sugar added to drinks, servings/week	-0.8 (-6.9, 5.3)	-2.5 (-11.1, 6.0)	-3.6 (-12.4, 5.2)
Water, servings/week	-2.9 (-10.2, 4.4)	4.1 (-5.5, 13.6)	4.7 (-2.4, 11.8)
Total physical activity, hours/week	-9.8 (-29.1, 9.5)	-12.0 (-34.2, 10.1)	-15.3 (-35.5, 4.9)
Total sleep time, hours/day	0.3 (-0.2, 0.8)	0.3 (-0.4, 1.0)	0.0 (-0.5, 0.4)
Total screen time, hours/week	-0.2 (-4.5, 4.1)	-2.3 (-7.5, 2.9)	-3.6 (-6.9, -0.4)

*Adjusted for child age, change in age from baseline to 3 months, sex, BMI z-score, and total physical activity at baseline; maternal education and occupation; and season of enrollment.

Behavioral models additionally adjust for baseline value of behavior of interest.

All models corrected for clustering by clinic.

### Feasibility

#### Scope and compliance

Of the 3095 children screened, 1406 (45%) were eligible, and 306 (22%) agreed to participate (Figure 1). To encourage attendance at the 3 month follow-up visits we called participants up to 10 times (Intervention mean = 2.6; Usual care mean = 3.2). Enrollment began in March 2012. Due to low participation, in August 2012 we began to offer reimbursement for transportation to the clinic. In November 2012 we began conducting drop-in home visits for participants who requested them or whom we were unable to contact by phone (Intervention n = 30; Usual care n = 29). After these changes, participation in 3 month follow-up visits rose from 50% to 75% in both groups. At the 6 month follow-up visits we again called participants up to 9 times (Intervention mean = 1.9; Usual care mean = 1.7) and conducted drop-in home visits (Intervention n = 29; Usual care n = 38).

We aimed for intervention families to attend all 6 of the weekly educational workshops, but only 52% (88 of the 168 who agreed to participate) attended ≥ 1 educational session (405 sessions attended in total). The total number of expected attendances at educational sessions was 1008 (168 participants attending 6 sessions each). Thus, compliance in the intervention group was 40% (405/1008) of total expected attendances. However, of the 88 receiving any intervention content, 67% (59/88) attended 5-6 of the intended 6 workshops.

At baseline, participants in the intervention group reported that transport to the clinic took mean (SD) 28 (16) minutes and cost $1.27 (1.19) one-way per person. Participants often brought several family members with them, multiplying the cost of transportation for the family. From the start of the study in March 2012 until August 2012 we did not offer reimbursement for transportation. From August 2012 until the end of follow-up in April 2013 we reimbursed transportation costs as an incentive for participation. The mean (SD) number of workshops participants attended rose from 2.03 (2.54) before this change to 3.04 (2.54) after this change, and percentage of participants attending any workshops rose from 43% to 68%.

#### Acceptability

The 69 intervention participants who attended educational workshops and completed a satisfaction survey at the 6 month follow-up visit reported that the total time involved in attending their last educational session was 3.01 (1.45) hours, and cost $2.35 (2.01) one-way per person for transportation alone and $1.81 (2.73) for other expenses.

Of the 69 intervention participants completing a satisfaction survey, 90% were “very satisfied” with the intervention and 100% would recommend the intervention to friends or family. No participant reported that the intervention diminished their satisfaction with their primary care clinic, and 83% reported their satisfaction with the primary care clinic had increased. We also asked participants how helpful each of intervention components was in achieving each of their behavioral goals, Table 5. Over 90% of participants reported that examples of appropriate portion sizes and learning to read nutrition labels and packages showing the quantities of sugar, fat and sodium that are contained in processed foods helped them “a lot” in reducing consumption of sugar-sweetened beverages and sweets, fried foods and packaged snacks. Sample recipes and the healthy eating plate were popular strategies for increasing fruits and vegetables. Across intervention topics, over 90% of participants reported that the in person group educational workshops with the study nutritionist helped them “a lot” in changing each of the targeted behaviors; while less helpful to parents than other strategies, over 80% of participants reported that the parent manual and calendar to log behavioral goals helped them “a lot”.

**Table 5:** **Helpfulness of intervention components reported by 69 of 168 parents participating in *Creciendo Sanos***

	**N (%)**			
	**Yes**	**No**	**Unsure**	
**Would you recommend the *Creciendo Sanos *** **educational sessions to friends and family?**	69 (100)	0 (0)	0 (0)	
	Very satisfied	Somewhat satisfied	Somewhat unsatisfied	Very unsatisfied
**In general, how satisfied are you with your participation in *Creciendo Sanos*?**	62 (90)	1 (1)	0 (0)	6 (9)
	Increased satisfaction with the clinic	Diminished satisfaction	No impact on satisfaction	
**What do you think of the medical attention you and your child have received in the clinic since your participation began?**	57 (83)	0 (0)	12 (17)	
	Not much	Some	A lot	
Average helpfulness of primary intervention components across targeted behaviors^*^				
Educational sessions with the nutritionist	2 (1)	20 (5)	362 (94)	
Parent obesity prevention manual	4 (1)	45 (12)	337 (87)	
Calendar to record changes in target behaviors	13 (3)	44 (11)	327 (85)	
***How much did each of the following help you to:***				
**Reduce sugar-sweetened beverages?**				
Labels demonstrating the quantity of sugar in beverages	1 (1)	6 (9)	62 (90)	
Examples of appropriate portion sizes for sweet beverages for children	0 (0)	5 (7)	64 (93)	
**Increase fruits and vegetables?**				
Healthy eating plate	0 (0)	3 (4)	66 (96)	
Sample healthy recipes for children’s meals	0 (0)	2 (3)	67 (97)	
**Reduce sweets, fried foods and packaged snacks?**				
Labels and packages showing sugar, fat and sodium contained in fried foods and sweets	0(0)	7(10)	62(90)	

*Results sum responses across all behavioral goals because the educational sessions, parent manual and calendar were intervention components used to address every behavioral goal. Other educational activities (e.g. learning to read nutrition labels to reduce added sugar in diet) were unique to specific goals.

## Discussion

In this pilot of a childhood obesity prevention intervention among preschool children in Mexico City, intervention children increased vegetable consumption at 3 months; we did not detect other intervention effects on diet, activity, or BMI at 3 or 6 months. Satisfaction among families that completed the intervention workshops was high, and we observed greater intervention effects among families with greater attendance at group educational workshops within the first 3 months.

Creciendo Sanos is the first obesity-specific prevention intervention for preschool children in IMSS primary care clinics. Given the high rates of overweight and obesity among Mexican preschoolers, and the broad reach of the IMSS primary care clinic and childcare networks, nationwide dissemination of effective strategies to improve the diet and physical activity through IMSS facilities has the potential for broad impact. The Creciendo Sanos pilot provides critical lessons learned for future obesity prevention interventions in IMSS clinics, including barriers to participation and strategies to change behavior to reduce risk of overweight and obesity. The intervention included the novel use of motivational interviewing techniques in a group setting and employed other strategies deemed best practices for childhood obesity prevention in recent reviews of the literature: parent engagement and role modeling; a limited number of clear and simple strategies (Figure 2) targeting both diet and physical activity in a combined intervention; and making healthy food and drink items (e.g. fruits and vegetables) available to children by preparing and tasting them during workshops [31-33].

Two prior, published interventions have provided nutritional counseling to already obese youth in Mexican health-care settings. One nonrandomized clinical trial delivered nutritional counseling to 40 obese youth aged 6 to 16 years showed reductions in BMI and improvements in lipid profiles [25]. The second study was a randomized controlled trial in a single primary care unit in Northern México which engaged 77 obese youth aged 9 to 17 years in a 12-month intervention. The intervention included a behavioral curriculum (weekly dietary advice for 3 months and monthly counseling thereafter); this trial found a -1.2 kg/m^2^ reduction in BMI [24]. In the Creciendo Sanos pilot we did not observe reductions in BMI at 3 or 6 months, potentially due to the shorter follow-up period, younger age of the participants and lower intensity of the intervention (6 weekly group educational sessions), as well as the low adherence in the intervention group. Another potential reason for the null effect on BMI is that 44% of children were normal weight according to WHO classifications at baseline (children with BMI z-score > 3 were ineligible) [34]. It is possible that families of obese children have greater motivation to make behavioral changes that decrease BMI.

Adherence in the intervention group was not as high as anticipated. Only 88 (52%) of participants in clinics randomized to intervention received ≥1 educational workshop; of those, 59 (67%) received 5-6 of the intended 6 workshops. Once reimbursement for transportation cost to the clinics began, workshop attendance rose, suggesting that cost of transportation was a barrier to attendance. Further, educational workshops were held in the clinic during working hours, which was recorded as difficult for employed parents in staff contact logs. We provided free childcare for participating children and siblings at every educational workshop and held individualized make-up sessions at multiple times convenient to mothers to offset barriers to attendance. However, there may have been disadvantages to offering individualized sessions: field notes reveal that group workshops allowed parents to brainstorm, advise and encourage one another. Field notes also document that establishing rapport – among participants and between participants and study staff – was crucial to attendance and participant motivation to change, and that offering visual materials such as nutrition labels made content accessible to parents of varying education levels.

A systematic review of the literature addressing attendance at clinic-based obesity prevention educational sessions for mothers of young children found greater attendance when the same session was offered at multiple times; however, offering childcare did not appear to improve attendance [35]. The same systematic review also found better attendance when sessions were integrated with routine well-child visits: while Creciendo Sanos scheduling took into account mothers’ preferred times, workshops were held weekly for a six week period rather than aligned with routine clinic visits, which occur much less frequently [35]. Future interventions should consider maximizing adherence by offering workshops at times tailored to individual families’ schedules and existing clinic visits.

Similar to our findings of greater intervention effects with greater adherence, in High Five greater participation in intervention activities also predicted greater change in target behaviors [17]. A meta-analysis of available intervention trials to prevent pediatric obesity found small beneficial changes on the target behaviors and no significant effect on BMI compared with control [20]. Consistent with our finding, longer treatments (>6 months) achieved larger reductions in sedentary activity and BMI than shorter trials, which were more effective in reducing unhealthy dietary behaviors [36]. Future research is needed to determine what degree of reinforcement and maintenance is needed to translate the short-term changes (such as those observed in this pilot) into sustained improvements in diet and physical activity that influence long-term weight trajectories.

With some behaviors, we found greater intervention effects with greater attendance at interventions workshops. Improving attendance required strategies (i.e., phone calls, home visits, and individual make-up workshops) that could be challenging to implement in Mexican health institutions given institutional processes and the staff and economic resources required. Additional strategies not employed in this intervention could be home visiting as a primary intervention delivery strategy rather than solely a retention strategy or better-aligning individual intervention visits with existing medical appointments, though this may impact the intensity and duration of intervention activities; another strategy is through specific incentives. In Mexico, the program *Oportunidades, “*Opportunities,” is a conditional cash transfer program designed to break the cycle of poverty. *Oportunidades* promotes children’s school attendance and the attendance of both mother and child to preventive health care services. This program may serve as a precedent and point of reference for effective strategies to motivate mothers and caregivers’ attendance in interventions aimed at preventing obesity [37]. Within IMSS, programs like these are not without precedent: for example, IMSS already provides financial support for patients traveling from one city to another to receive medical care, home visits for directly observed therapy for tuberculosis patients, and shuttles for patients with chronic renal failure attending dialysis appointments.

We did not administer a formal survey asking participants about reasons for non-participation, poor compliance and lack of behavioral change; however, from contact logs and field notes, parents expressed mainly the following reasons: work schedules, lack of time, domestic responsibilities such as caring for family members, lack of interest in the study and not needing the information offered. In many cases, eligible families who decided not take part in the study gave no reason for refusing or cited lack of interest in the health problem of obesity. In formative research for this trial, we found that a majority of parents of overweight and obese children acknowledged their child’s overweight status when informed, but did not acknowledge the health consequences [16]. Further research is needed to determine what health messages and approaches could be effective at engaging families in primary-care based obesity prevention interventions.

### Limitations

As this was a pilot study, we limited the study to 4 clinics; in cluster randomized trials, 4 clusters are not enough to guarantee balance of individual characteristics at baseline. However, adjusted and unadjusted results were similar, suggesting that any imbalance in observed (or unobserved) characteristics did not affect inferences. Despite adaptive retention strategies including drop-in home visits for participants who could not be contacted by phone (Additional file 1: Table S2), 35% of families did not complete follow-up at 3 months, our primary endpoint. To overcome the limitation of these missing data, we performed multiple imputations, but higher retention and more detailed qualitative information on reasons for non-participation would be preferred. As discussed above, adherence to intervention activities was not optimal; this could have resulted in weaker effects than if adherence were higher.

Behavioral outcomes relied on parental report rather than objective measures. It is possible that parents could exaggerate self-reported improvements in behaviors, but in any case we did not observe major effects on behavior change in intention to treat analyses.

## Conclusion

In summary, the Creciendo Sanos intervention did not change overall diet or physical activity-related behaviors, although we observed greater changes in some behavior with greater adherence to the intervention. Though non-compliance and loss to follow-up were important limitations, participating families were highly satisfied with the intervention.

Lessons learned for future interventions include improving access and comprehensiveness to increase participation and adherence. When childhood obesity prevention interventions are implemented on a broad scale in the existing health system, interventionists should consider activities to improve access. Creciendo Sanos activities that could be replicated in future interventions include increasing retention via drop-in home visits; increasing adherence by providing reimbursement of transportation costs and offering workshops at times tailored to individual families’ schedules given domestic and professional responsibilities; and increasing inclusiveness by establishing rapport with and among families and including ample visual materials to engage parents of all education levels (e.g. instruction on how to interpret food labels and nutritional content of processed foods). A comprehensive intervention takes into account the sociocultural context and is complemented by strategies to motivate participation, not only effective health communication to inform the population about obesity’s causes and consequences, but also the institutionalization of obesity prevention interventions as a component of health care that patients are accustomed to receiving, as happens with antenatal care and family planning.

In Mexico, health policies to prevent overweight and obesity are gaining momentum and advocacy in the public and private sectors is increasing. Since 2006 the Ministry of Health has promoted a national policy for obesity prevention, which became the National Strategy for Prevention and Control of Overweight, Obesity and Diabetes [38]. This strategy sets out three pillars: public health, health care, and health regulation and fiscal policy. Since 2014, the increase in the tax for sugar-sweetened beverages is in effect and the education sector has banned sodas and unhealthy food in schools. Furthermore, there is an effort to strengthen regulations for food and beverage marketing to children and innovations that make front-of-pack labeling systems more understandable to the public. Though the efforts to tackle obesity range from public policies to specific programs, the need to further incentivize the population’s participation in individual-centered interventions is clear.

## Abbreviations

IMSS: Instituto Mexicano del Seguro Social; BMI: Body mass index; WHO: World Health Organization; ChiquitIMSS: Educational strategy for health promotion for children 3-6 years old; FFQ: Food frequency questionnaire; DVD: Digital versatile disc; S1: Session one; S2: Session two; S3: Session three; S4: Session four; S5: Session five; S6: Session six.

## Competing interests

The authors declare that they have no competing interests.

## Authors’ contributions

All authors contributed to the conceptualization and design of the intervention. MWG, RPC and ET obtained funding for the project. GOMA, GRQ and MABT managed the study staff and implemented the intervention. EMC and GOMA drafted and revised the manuscript. SRS, EMC and GOMA analyzed the data. MAGU, SFH, CH, JH, RPC and MWG provided input on early drafts. All authors read and approved the final manuscript.

## Pre-publication history

The pre-publication history for this paper can be accessed here:

http://www.biomedcentral.com/1471-2431/14/77/prepub

## Supplementary Material

Additional file 1: Table S1 Behavioral targets and measures used in *Creciendo Sanos*: a clinic-based intervention to prevent obesity in Mexico City preschool children. **Table S2.** Locating participants. **Figure S1.** Study Timeline. Click here for file
