# Supplementary material for: Feasibility and impact of Creciendo Sanos, a clinic-based pilot intervention to prevent obesity among preschool children in Mexico City
Source: BMC Pediatr. 2014 Mar 20;14:77. doi: 10.1186/1471-2431-14-77 (PMC3999907; doi:10.1186/1471-2431-14-77)
Supplement: Additional file 1: Table S1 — Behavioral targets and measures used in Creciendo Sanos: a clinic-based intervention to prevent obesity in Mexico City preschool children. Table S2. Locating participants. Figure S1. Study Timeline. [file 1471-2431-14-77-S1.docx]

| **Table S1. Behavioral targets and measures used in *Creciendo Sanos*:**  **a clinic-based intervention to prevent obesity in Mexico City preschool children** | | | | |
| --- | --- | --- | --- | --- |
| **Educational Workshop** | **Intervention Goals** | **Session Activities** | **Home Activities** | **Measures** |
| **Session 1**  Group ice breakers and introductions.  Participant expectations regarding nutrition and physical activity sessions. | Presentation of educational session curriculum and discussion of group expectations. | Record participant expectations for educational sessions.  Propose a dinner menu, using magazine clippings and food cards provided. | Create a recipe. | Verbal expectations regarding educational workshops and commitment to participate. |
| **Session 2**  Healthy Eating Plate: how to include all three groups in every meal and create healthy recipes children will like.  Sugar content of popular processed foods. | Increase consumption and availability of:   - Plain water - Fruits and vegetables - Whole grains   Limit consumption of:   - Refined carbohydrates - Fatty and fried foods - Sugary drinks | Using sample foods, propose a healthy children’s meal.  Put processed, commercial foods in order according to fat and added sugar content. | Create a recipe.  Color in healthy eating plate.  Fill in calendar log with behavioral goals for child nutrition and physical activity.  Recommendations: Combine elements from three food groups at every meal, choose whole grains, fruits, vegetables and plain water; decrease consumption of sugar-sweetened beverages and fried and fast foods and packaged snacks | Number and frequency of portions of foods consumed in a day (food frequency questionnaire), including the number and portions of processed foods. |
| **Session 3**  Age-appropriate portion sizes for children.  Shopping list based on a healthy menu plan. | Provide information about appropriate portions sizes for children of different ages.  Increase consumption and availability of:   - Plain water - Fruits and vegetables - Whole grains | Create a daily child menu with appropriate distribution of portions at breakfast, lunch, dinner and two snacks. | Complete weekly menus.  Create a shopping list for needed ingredients for weekly menu.  Fill in the calendar log with behavioral goals for child nutrition and physical activity. | Number and frequency of portions of foods consumed in a day (food frequency questionnaire). |
| **Session 4**  Habits and routines: formation of and influence on child behaviors.  Food purchasing.  Implementing behavioral change. | Recognizing dietary and activity habits that could lead to overweight.  Establishing healthy dietary and activity habits.  Increasing the availability of healthy food at home. | Record children’s diet and activity the previous day.  Record foods typically purchased for household.  Revise recipes previously recorded. | Use the shopping list.  Fill in calendar log with behavioral goals for child nutrition and physical activity. | Number and frequency of portions of foods consumed in a day (food frequency questionnaire).  Time spent in physical activity and inactivity (activity questionnaire). |
| **Session 5**  Importance of monitoring child growth.  Identifying BMI on child growth charts.  Participating in physical activity with children. | Understanding how to evaluate healthy growth using height and weight measurements.  Increasing children’s physical activity.  Decreasing screen time. | Calculate children’s BMI using height and weight and identify weight status on a growth chart.  Make a poster to promote children’s physical activity. | Complete a poster promoting physical activity for child/ family.  Fill in calendar log with behavioral goals for child nutrition and physical activity. | Time spent in physical activity and inactivity (activity questionnaire). |
| **Session 6**  Ideas for active play.  Review of overweight: causes and consequences.  Plan to maintain behavioral changes. | Increasing children’s physical activity.  Decreasing screen time.  Implementing dietary and activity habits that help children maintain a healthy weight. | Present the physical activity promotion posters made in the prior session.  Discuss the consequences of childhood overweight and obesity. | Maintain changes implemented over the course of the sessions in children’s nutrition and physical activity. | Number and frequency of portions of foods consumed in a day (food frequency questionnaire).  Time spent in physical activity and inactivity (activity questionnaire).  Measurement of child height and weight at 3 and 6 month follow-up. |


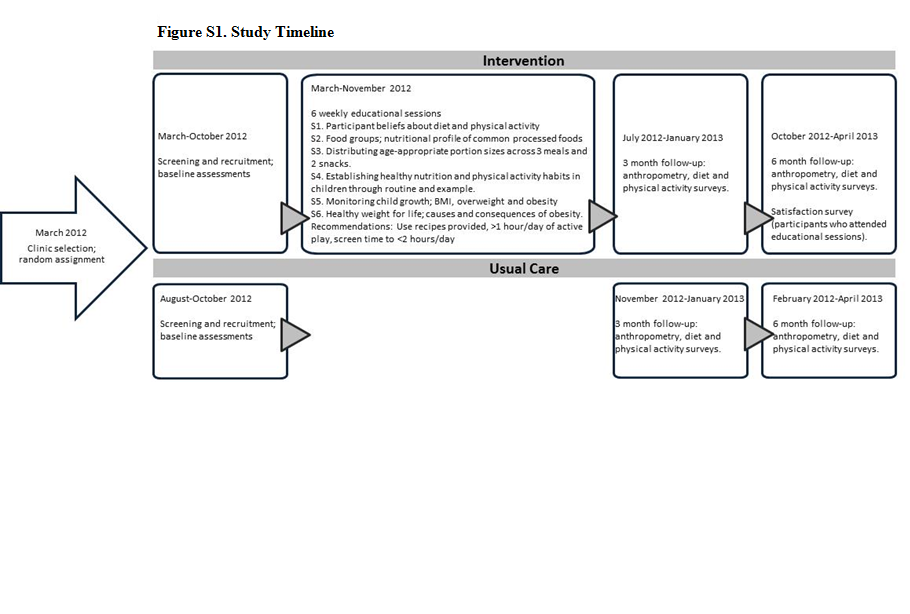


| **Table S2. Locating participants** | | | | | | | | | | |
| --- | --- | --- | --- | --- | --- | --- | --- | --- | --- | --- |
|  | Educational sessions | | 3 month follow-up | | | | 6 month follow-up | | | |
|  | Intervention | | Intervention | | Control | | Intervention | | Control | |
|  |  | | **Mean(range)** | | | | | | | |
| **Reminder phone calls** | 5 (0-8) | | 2.6 (0-10) | | 3.2 (0-10) | | 1.9 (0-7) | | 1.7 (0-9) | |
|  |  |  |  | | | | | | | |
|  |  |  | **N(%)** | | | | | | | |
| **Located by phone?** |  |  |  |  |  |  |  |  |  |  |
| Yes |  |  | 118 (70.2) | | 90 (65.2) | | 123 (73.2) | | 92 (66.7) | |
| No |  |  | 50 (29.8) | | 48 (34.8) | | 45 (26.8) | | 46 (33.3) | |
| **Follow-up visit scheduled once located by phone?** |  |  |  |  |  |  |  |  |  |  |
| Yes, in clinic |  |  | 88 (52.4) | | 80 (58.0) | | 89 (72.4) | | 73 (79.4) | |
| Yes, at home |  |  | 6 (3.6) | | 8 (4.6) | | 14 (11.4) | | 15 (16.3) | |
| No |  |  | 24 (20.3) | | 2 (2.2) | | 20 (16.3) | | 4 (4.4) | |
| **Follow-up visit completed once scheduled by phone?** |  |  |  |  |  |  |  |  |  |  |
| Yes, in clinic |  |  | 78 (88.6) | | 63 (78.8) | | 81 (91.0) | | 60 (82.1) | |
| Yes, at home |  |  | 6 (100) | | 7 (7.5) | | 13 (92.8) | | 14 (93.3) | |
| **Was participant eligible for drop-in home visit (unable to contact by phone)?** |  |  |  |  |  |  |  |  |  |  |
| Yes |  |  | 30 (17.9) | | 64 (46.4) | | 29 (17.3) | | 28 (20.3) | |
| No |  |  | 138 (82.1) | | 74 (53.6) | | 139 (82.7) | | 110 (79.7) | |
| **Was follow-up completed at drop-in home visit?** |  |  |  |  |  |  |  |  |  |  |
| Yes |  |  | 14 (46.7) | | 29 (45.3) | | 11 (37.9) | | 24 (85.7) | |
| No |  |  | 16 (53.3) | | 35 (54.7) | | 18 (62.1) | | 4 (14.3) | |
| **Total visits completed** |  |  |  |  |  |  |  |  |  |  |
| In clinic |  |  | 79 (79.8) | | 64 (62.7) | | 85 (78.0) | | 61 (61.6) | |
| In home |  |  | 20 (20.2) | | 38 (37.3) | | 24 (22.0) | | 38 (38.4) | |
